# Risk Factors of Lymph Node Metastasis and Its Prognostic Significance in Early Gastric Cancer: A Multicenter Study

**DOI:** 10.3389/fonc.2021.649035

**Published:** 2021-10-13

**Authors:** Jinfeng Wang, Liang Wang, Sha Li, Fei Bai, Hailong Xie, Hanguo Shan, Zhuo Liu, Tiexiang Ma, Xiayu Tang, Haibing Tang, Ang Qin, Sanlin Lei, Chaohui Zuo

**Affiliations:** ^1^ Department of Gastroduodenal and Pancreatic Surgery, Translational Medicine Research Center of Liver Cancer, Laboratory of Digestive Oncology, The Affiliated Cancer Hospital of Xiangya School of Medicine and Hunan Cancer Hospital (Hunan Cancer Institute), Central South University, Hunan Province Key Laboratory of Virology (Tumor Immunity), Changsha, China; ^2^ Graduates School, University of South China, Hengyang, China; ^3^ Graduates School, Department of Gastrointestinal Surgery of Second Affiliated Hospital, Cancer Research Institute, University of South China, Hengyang, China; ^4^ The Third Department of General Surgery, The Central Hospital of Xiangtan City, Xiangtan, China; ^5^ Department of General Surgery, Yongzhou Central Hospital, Yongzhou, China; ^6^ Department of General Surgery, People Hospital of Qiyang County, Yongzhou, China; ^7^ Department of General Surgery, The Second Xiangya Hospital of Central South University, Changsha, China

**Keywords:** early gastric cancer (EGC), lymph node metastasis (LNM), risk factors, lymph node dissection, depth of invasion

## Abstract

**Background:**

Early gastric cancer (EGC) is invasive gastric cancer that invades no deeper than the submucosa, regardless of lymph node metastasis (LNM). It is mainly treated by surgery. Recently, the resection range of EGC has been minimized, but cancer recurrence and overall survival in some patients should be given high status. LNM is an important indicator of prognosis and treatment in gastric cancer. The law of the number and location of metastatic lymph nodes in EGC is not yet clear. Therefore, we aimed to identify the risk factors of LNM in radically resected EGC and guide treatment.

**Methods:**

The clinicopathological factors of 611 patients with EGC were retrospectively analyzed in six hospitals between January 2010 and December 2016. The relationship between clinicopathological factors and LNM, as well as their prognostic significance, were analyzed by univariate and multivariate analyses.

**Results:**

The rate of LNM was 20.0% in the 611 EGC patients. The depth of invasion, differentiation type, tumor diameter, morphological ulceration, and lymphovascular invasion were independent risk factors for LNM (*P*<0.05) by logistic regression analysis. Tumor location in the proximal third of the stomach and morphological ulceration were significant factors for group 2 LNM. Moreover, the 5-year survival rate was 94.9% for patients with no positive nodes, 88.5% for patients with 1-2 positive nodes, 64.3% for patients with 3-6 positive nodes, and 41.8% for patients with >6 metastatic nodes. Interestingly, the 7-year risk of relapse diminished for patients with no LNM or retrieved no less than 15 lymph nodes.

**Conclusions:**

Fifteen lymph node dissection and D2 radical operation are the surgical options in case of high risk factors for LNM. Extended lymph node dissection (D2+) is recommended for morphological ulceration or disease located in the proximal third of the stomach due to their high rate of group 2 LNM. Furthermore, LNM is a significant prognostic factor of EGC. Moreover, lymph nodes can also play a significant role in the chemotherapeutic and radiotherapy approach for non-surgical patients with EGC.

## Introduction

Gastric cancer (GC) is one of the most common malignancies of the gastrointestinal tract and has a serious impact on public health. Furthermore, GC is the fifth malignancy and the third-most common cause of cancer-related deaths worldwide (1). The prognosis of GC is poor, as the 5-year survival rate is <30% (2). This is mostly because most cases are diagnosed in the advanced stage of the cancer that presents with metastases, high intratumor heterogeneity, and chemotherapeutic resistance, thereby leading to overall poor outcomes (2). Presently, the treatment for GC depends on its stages at diagnosis. Early stages can be cured endoscopically or surgically; intermediate stages require neoadjuvant chemotherapy to improve tumor status for subsequent resection; whereas late stage GC is mainly treated non-surgically by chemotherapy and radiotherapy approach (2, 3). Early gastric cancer (EGC) is defined as a lesion confined to the mucosa or the submucosa, irrespective of the presence of regional lymph node metastases (LNM). The early detection of GC has increased in China because of the national early cancer screening policy (4). The prognosis of EGC is satisfactory, with the 5-year survival rate tending to be >90% (5). Kunisaki et al. (6) analyzed 1,169 patients with EGC who underwent surgery: 1,052 patients without LNM had a 5-year survival rate of 99.1%, and 117 patients with LNM had a 5-year survival rate of 90.8%. Recently, the resection range of EGC seems to be minimized, however, the cancer recurrence and overall survival (OS) in some patients should be given high status. Many factors such as LNM, depth of wall invasion, macroscopic type, and differentiation type affect the prognosis of EGC. The significantly prognostic factor in EGC is LNM (7). Several risk factors for LNM in EGC, such as tumor size, invasion depth, ulceration, histological types, and lymphovascular invasion, have been reported in previous studies (8). Lymph node metastasis is an important disease feature that affects the prognosis and determines the extent of lymph node dissection (9). The number of metastatic lymph nodes (MLNs) is reportedly related to mortality risk (6, 7, 10). Patients with MLNs had a relatively higher recurrence rate and poorer survival rate than those with no MLNs (11). Therefore, risk factors for LNM should be considered when choosing a surgery scheme for patients with EGC.

In this study, we retrospectively evaluated the distribution of LNM in a six-center cohort including 611 patients with EGC. The relationship between clinicopathological factors and LNM, the extent of LNM in EGC, and their prognostic significance were analyzed by univariate and multivariate analyses. By analyzing the clinical characters of EGC, investigating the rate of LNM, and clarifying the risk factors of LNM, we aimed to provide a basis for choosing the optimal surgical scheme and determining the appropriate range of lymph node dissection.

## Methods

### Patients

We retrospectively reviewed EGC cases that had complete clinical and pathological data and underwent curative gastrectomy with lymphadenectomy in the Department of Surgery in the six hospitals between November 2010 and December 2016. A total of 611 patients (384 males and 227 females, mean age: 55 (22–85) years) were reviewed in this research: 363 patients in Hunan Province Cancer Hospital, 160 patients in the Second Xiangya Hospital of Central South University, 30 patients in the Second Affiliated Hospital of South China University, 26 patients in the Central Hospital of Xiangtan City, 22 patients in Yongzhou Central Hospital and 10 patients in People Hospital of Qiyang county. All patients were pathologically diagnosed with EGC and received consultation by the Multiple Disciplinary Team (MDT) at each center. Radical resection was then performed in all patients who did not undergo or did not wish to undergo neoadjuvant chemotherapy. Data on clinical parameters such as age, sex, and cancer embryonic antigen (CEA) level before the operation; postoperatively confirmed pathological parameters including depth of invasion, differentiation type, and lymphovascular invasion (LVI); and macroscopic features such as macroscopic type, tumor diameter, location, and morphological ulceration were collected retrospectively. Moreover, the number and station of MLNs were also reviewed in detail.

Early gastric cancer was more frequently located in the distal third of the stomach (lower cancer, 284 cases, 46.5%) than in the proximal (upper cancer, 86 cases, 14.1%) or middle third (middle cancer, 241 cases, 39.4%). The average number of retrieved lymph nodes was 17 (9–32). Because at least 15 retrieved lymph nodes are required for better staging and lower risk of recurrence of EGC (12), we divided the retrieved lymph nodes into two groups: <15 retrieved lymph nodes and ≥15 retrieved lymph nodes. Details of EGC patients are shown in **Table 1**.

**Table 1:** Demographics of 611 patients with early gastric cancer.

Clusters	Patients (%)
Sex	
Male	384 (62.8%)
Female	227 (37.2%)
Age	
>60	215 (35.2%)
≤60	396 (64.8%)
Depth of invasion	
Mucosa	205 (33.6%)
Submucosa	406 (66.4%)
Differentiation type	
Well differentiated cancer	327 (53.5%)
Undifferentiated cancer	284 (46.5%)
Lesion location	
Lower cancer (L)	284 (46.5%)
Middle cancer (M)	241 (39.4%)
Upper cancer (U)	86 (14.1%)
Tumor diameter	
<1 cm	108 (17.7%)
1–3 cm	287 (47.0%)
>3 cm	216 (35.3%)
Macroscopic type	
Elevated type	116 (19.0%)
Flat type	153 (25.0%)
Depressed type	342 (56.0%)
Morphological ulceration	
No	269 (44.0%)
Yes	342 (56.0%)
LVI	
No	566 (92.6%)
Yes	45 (7.4%)
Serum CEA	
<5 ng/ml	560 (91.7%)
≥5 ng/ml	51 (8.3%)
LNM	
No	489 (80.0%)
Yes	122 (20.0%)
retrieved LN	
<15	218 (35.7%)
**≥**15	393 (64.3%)

LVI, lymphovascular invasion; CEA, Carcinoembryonic antigen; LNM, lymph node metastasis; LN, lymph nodes.

### Surgery

Among the 611 patients, 251 underwent open radical gastrectomy, cases underwent laparoscopic-assisted radical gastrectomy; D1 and D2 lymph node dissection were performed concurrently. The choice of the surgical scheme and the division of lymphadenectomy scope were in line with the 15th Japanese Classification of Gastric Carcinoma (13). Routinely, the greater omentum, anterior lobe of the transverse mesocolon, and pancreatic capsule were incised. The distance between the incision line and the outer edge of the cancer was determined by Borrmann classification and found to be 4–7 cm. The distances in the case of Borrmann types I, II, III, and IV cancers were 2, 3–4, 5–6, and 6–7 cm, respectively. There were 352 cases of distal gastrectomy, 216 cases of proximal gastrectomy, and 43 cases of total gastrectomy. Specifically, Roux-en-Y esophagojejunostomy was used for the reconstruction of the alimentary tract following total gastrectomy. Billroth’s operations I and II were used for the reconstruction of the alimentary tract following distal partial gastrectomy in 315 and 37 cases, respectively.

### Pathological Examination and Data Collection

The resected specimen was dissected to observe for morphological ulceration in the tumor and calculate the tumor size according to its maximum surface diameter. According to the classification scheme formulated by the Japanese Endoscopy Society, the macroscopic type was classified as elevated (type  I  or II a), flat (II b), or depressed (II c or III). Histologic types were divided into differentiated type (papillary adenocarcinoma, tubular adenocarcinoma, and high-grade differentiated adenocarcinoma) and undifferentiated type (low-differentiated adenocarcinoma, undifferentiated adenocarcinoma, mucinous adenocarcinoma, and signet ring cell carcinoma) based on the criteria of the World Health Organization (WHO). Each lymph node was embedded in paraffin and at least two sections were performed. Hematoxylin-eosin (HE) staining was used to determine whether lymph nodes were metastatic. MLNs were classified into two groups based on the Japan Gastric Cancer Association (JGCA) classification: group 1, metastasis only in the first-tier lymph nodes; and group 2, metastasis in lymph nodes in the second-tier and over, with or without first-tier metastasis. Gastric cancer specimen processing, pathological diagnosis, assessment of diagnostic criteria, and lymph node classification were performed based on the 15th Japanese Classification of Gastric Carcinoma (13) and the 8th edition of gastric cancer TNM staging system (14).

### Follow-Up

Patients with EGC were followed up regularly after a radical operation. The last follow-up date was July 30, 2020. The patients were followed up every 6 months for the first 3 years after surgery, and then once a year until death or loss to follow-up. The follow-up information, including the time of patient relapse or death, was obtained from hospital information systems and the patients or their relatives. Overall survival (OS) was calculated from the date of pathological diagnosis to death or the last date of follow-up.

### Statistical Analysis

The data were statistically processed using SPSS 22.0 software (IBM Corporation, Armonk, NY, USA). The relationship between clinicopathological characteristics and the status of lymph node metastasis was analyzed by the chi-square test. Univariate and multivariate logistic regression models were used to estimate predictors of LNM. The survival curve in the function of lymph node status was traced using the Kaplan–Meier method. The difference between curves was tested using the log-rank test. The Kaplan–Meier method was also used to estimate the 5-year survival rate and 7-year relapse rate of each subgroup of the clinicopathological variable. The influence of the clinicopathological variable on the 5-year survival rate and 7-year relapse rate was examined using the chi-square test. Multivariate Cox regression analysis was used to estimate independent prognostic factors.

## Results

### Clinicopathological Features and LNM

The mean number of retrieved lymph nodes from the 611 patients with EGC was17 (9–32); in 64.3% patients at least 15 lymph nodes were retrieved. Lymph node metastasis was found in 122 patients, and the rate of LNM was 20.0% (122/611). Among them, the rate of LNM was 20.3% (78/384) for male and 19.4% (44/227) for female patients; 19.1%(41/215) for age>60 years and 20.7%(81/396) for age ≤ 60 years; 14.1% (29/205) for submucosal cancer and 22.9% (93/406) for mucosal cancer; 14.4% (47/327) for well differentiated cancer and 26.4% (75/284) for undifferentiated cancer; 18.7% (53/284) for lower cancer (L), 19.1% (46/241) for middle cancer (M) and 26.7% (23/86) for upper cancer (U); 14.8% (16/108) for tumor size <1 cm, 17.8% (51/287) for tumor size between 1 and 3 cm, and 24.8% (55/216) for tumor size >3 cm; 14.7% (17/116) for elevated type, 15.7% (24/153) for flat type, and 23.7% (81/342) for depressed type; 42.2% (19/45) for LVI and 18.2% (103/566) for no LVI;19.5% (109/560) for serum CEA <5 ng/mL and 25.5% (13/51) for serum CEA ≥5 ng/mL; 17.0% (37/218) for retrieved lymph nodes <15 and 21.7% (85/308) for retrieved lymph nodes ≥15, respectively (**Table 2**).

**Table 2:** The relationship between clinicopathological characteristics and status of lymph node metastasis in EGC.

Clusters	LNM (-) (%)	LNM (+) (%)	*P*
Sex			0.781
Male	306 (79.7%)	78 (20.3%)	
Female	183 (80.06%)	44 (19.4%)	
Age			0.683
>60	174 (80.9%)	41 (19.1%)	
≤60	315 (79.5%)	81 (20.5%)	
Depth of invasion			0.011
Mucosa	176 (85.9%)	29 (14.1%)	
Submucosa	313 (77.1%)	93 (22.9%)	
Differentiation type			<0.001
Well differentiated	280 (85.6%)	47 (14.4%)	
Undifferentiated	209 (73.6%)	75 (26.4%)	
Lesion location			0.236
Lower cancer	231 (81.3%)	53 (18.7%)	
Middle cancer	195 (80.9%)	46 (19.1%)	
Upper cancer	63 (73.3%)	23 (26.7%)	
Tumor diameter			0.036
<1 cm	92 (85.2%)	16 (14.8%)	
1–3 cm	236 (82.2%)	51 (17.8%)	
>3 cm	161 (74.5%)	55 (25.5%)	
Macroscopic type			0.032
Elevated type	116 (19.0%)	17 (14.7%)	
Flat type	153 (25.0%)	24 (15.7%)	
Depressed type	342 (56.0%)	81 (23.7%)	
Morphological ulceration			0.010
No	228 (84.8%)	41 (15.2%)	
Yes	261 (76.3%)	81 (23.7%)	
LVI			<0.001
No	463 (81.8%)	103 (18.2%)	
Yes	26 (57.8%)	19 (42.2%)	
Serum CEA			0.303
<5 ng/ml	451 (80.5%)	109 (19.5%)	
≥5 ng/ml	38 (74.5%)	13 (25.5%)	
Retrieved LN			0.168
<15	181 (83.0%)	37 (17.0%)	
**≥**15	308 (78.4%)	85 (21.7%)	

LVI, lymphovascular invasion; CEA, Carcinoembryonic antigen; LNM, lymph node metastasis; LN, lymph nodes.

There were 61 cases (50.0%) with one positive node, 25 (20.5%) with two positive nodes, 19 (15.6%) with 3–5 positive nodes, and 17 (13.9%) with >6 metastatic nodes. The positive rate of lymph node was the highest in the N6 group (35.2%, 43/122), followed by the N3 group (27.0%, 33/122), N4d group (19.7%, 24/122), N7 group (18.9%, 23/122), N5 group (18.0%, 22/122), N9 group (17.2%, 21/122), N8a group (13.9%, 17/122), N1 group (6.6%, 8/122), N11p group (4.1%, 5/122) and N12a group (3.3%, 4/122). Further, 73.8% (90/122) patients who had only group 1 LNM and 26.2% (32/122) patients who had group 2 LNM. Interestingly, upper cancer and morphological ulceration are susceptible to group 2 LNM (*P*=0.033 and *P*=0.038, respectively). However, age, sex, depth of tumor invasion, differentiation type, tumor diameter, macroscopic type, size of tumor diameter, LVI, serum CEA, and the number of retrieved lymph nodes were not related to group 1 and 2 LNM (*P*> 0.050) (**Table 3**). As LNM is closely related to TNM stage, we further analyzed the relationship between group 1and 2 LNM and the TNM stage for the tumors in different size groups. The rate of group 2 LNM showed a trend for higher stage II–III than stage I for tumors sized >3cm (*P*=0.080) (**Supplementary Table 1**).

**Table 3:** The relationship between clinicopathological characteristics and group 1 and 2 lymph node metastasis in EGC.

Clusters	Group 1 LNM (%)	Group 2 LNM (%)	*P*
Sex			0.138
Male	61 (78.2%)	17 (21.8%)	
Female	29 (65.9%)	15 (34.1%)	
Age			0.742
>60	31 (75.6%)	10 (24.4%)	
≤60	59 (72.8%)	22 (27.2%)	
Depth of invasion			0.769
Mucosa	22 (75.9%)	7 (24.1%)	
Submucosa	68 (73.1%)	25 (26.9%)	
Differentiation type			0.574
Well differentiated	36 (76.6%)	11 (23.4%)	
Undifferentiated	54 (72.0%)	21 (28.0%)	
Lesion location			0.033
Lower cancer	42 (79.2%)	11 (20.8%)	
Middle cancer	36 (78.3%)	10 (21.7%)	
Upper cancer	12 (52.2%)	11 (47.8%)	
Tumor diameter			0.229
<1 cm	14 (87.5%)	2 (12.5%)	
1–3 cm	39 (76.5%)	12 (23.5%)	
>3 cm	37 (67.3%)	18 (32.7%)	
Macroscopic type			0.110
Elevated type	15 (88.2%)	2 (11.8%)	
Flat type	20 (83.3%)	4 (16.7%)	
Depressed type	55 (67.9%)	26 (32.1%)	
Morphological ulceration			0.038
No	35 (85.4%)	6 (14.6%)	
Yes	55 (67.9%)	26 (32.1%)	
LVI			0.564
No	77 (74.8%)	26 (25.2%)	
Yes	13 (86.4%)	6 (31.6%)	
Serum CEA			0.694
<5 ng/ml	81 (74.3%)	28 (25.7%)	
≥5 ng/ml	9 (69.2%)	4 (30.8%)	
Retrieved LN			0.226
<15	30 (81.1%)	7 (18.9%)	
**≥**15	60 (70.6%)	25 (29.4%)	

LVI, lymphovascular invasion; CEA, Carcinoembryonic antigen; LNM, lymph node metastasis; LN, lymph nodes.

### Univariate Analysis of LNM and Clinicopathological Factors

The depth of tumor invasion, differentiation type, macroscopic type, morphological ulceration, size of tumor diameter, and LVI were related to LNM (*P*< 0.050), but age, sex, and tumor location were not related to LNM (*P*> 0.050) (**Table 4**).

**Table 4:** Univariate analysis of lymph node metastasis and clinicopathological factors in EGC.

Risk factor	lymph node metastasis		
	OR	95% CI	*P*
Sex			0.781
Male	Reference		
Female	0.943	0.624-1.425	
Age			0.683
>60	Reference		
≤60	1.091	0.718-1.659	
Depth of invasion			0.011
Mucosa	Reference		
Submucosa	1.803	1.143-2.845	
Differentiation type			<0.001
Well differentiated	Reference		
Undifferentiated	2.138	1.424-3.209	
Lesion location			
Lower cancer	Reference		0.240
Middle cancer	1.028	0.663-1.594	
Upper cancer	1.591	0.906-2.794	
Tumor diameter			0.036
<1 cm	Reference		
1~3 cm	1.243	0.674-2.289	
>3 cm	1.964	1.064-3.625	
Macroscopic type			0.036
Elevated type	Reference		
Flat type	1.083	0.552-2.126	
Depressed type	1.807	1.020-3.201	
Morphological ulceration			0.010
No	Reference		
Yes	1.726	1.139-2.615	
LVI			<0.001
No	Reference		
Yes	3.285	1.751-6.161	
Serum CEA			0.305
<5 ng/ml	Reference		
≥5 ng/ml	1.415	0.729-2.749	
Retrieved LN			0.168
<15	Reference		
**≥**15	1.350	0.880-2.070	

LVI, lymphovascular invasion; CEA, Carcinoembryonic antigen; LNM, lymph node metastasis; LN, lymph nodes.

### Multivariate Analysis of LNM and Clinicopathological Factors

Because single-factor analysis could not control the confounding factors and may enhance or weaken the effect of some clinicopathological characteristics on the LNM of EGC, the factors with statistical significance in single factor analysis were further analyzed by multifactor logistic multivariate analysis. Univariate analysis showed that the LNM rate of tumor diameter 1–3 cm and tumor diameter <1 cm subgroups was not statistically significant. Thus, in the multivariate analysis, the tumor diameter 1–3 cm subgroup and tumor diameter <1 cm subgroup were combined into a tumor diameter ≤3 cm subgroup to improve the efficiency of statistical testing. Logistic multivariate analysis revealed that the depth of tumor invasion, differentiation type, tumor diameter, morphological ulceration, and LVI were independent risk factors for EGC lymph node metastasis (*P* < 0.050).

### Long-Term Outcomes and Survival Analysis

**Figure 1:**
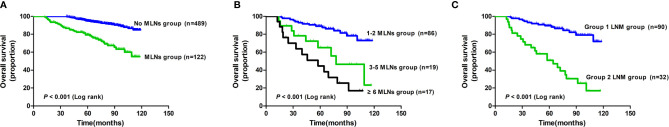
Survival Analysis of 611 EGC patients. **(A)** Survival analysis of groups with or without MLNs. **(B)** Survival analysis of groups based on the number of MLNs. **(C)** Survival analysis of groups based on group 1 and 2 LNM. MLNs, metastatic lymph nodes; LNM, lymph node metastasis.

There were 78 deaths (12.7%) during a median follow-up of 72.3 months (range: 12.5–118.8 months). The 1-, 3-, and 5-year survival rates were 100%, 100%, and 94.9% in the group with no MLNs, and 100%, 86.7%, and 81.1% in the MLNs group, respectively (**Figure 1A**). Furthermore, the 5-year survival rate was 88.5% for tumors with 1–2 positive nodes, 64.3% for tumors with 3–5 positive nodes, and 41.8% for tumors with >6 metastatic nodes (**Figure 1B**). Moreover, the OS of patients with group 1 LNM was better than that of patients with group 2 LNM (*P*< 0.001, **Figure 1C**). Univariate analysis revealed that the depth of tumor invasion, differentiation type, morphological ulceration, LVI, regional LNM, and retrieval of at least 15 lymph nodes were related to the 5-year survival rate (*P*< 0.05, **Table 5**), while age, sex, tumor location, tumor diameter, macroscopic type, and serum CEA were not related to the 5-year survival rate (*P* > 0.05, **Table 5**). Multivariate Cox regression analysis showed that regional LNM was the unique independent risk factor that affected the prognosis of patients with EGC (HR 5.157, 95%CI 3.216-8.268, *P*< 0.01).

**Table 5:** The relationship between clinicopathological characteristics and 5-year survival rate and 7-year relapse rate in EGC.

Clusters	Amount	5-year survival rate (%)	*P*	7-year relapse rate (%)	*P*
Sex			0.795		0.736
Male	384	91.9%		14.8%	
Female	227	92.5%		15.9%	
Age			0.328		0.864
>60	215	90.7%		14.9%	
≤60	396	92.9%		15.4%	
Depth of invasion			0.024		0.215
Mucosa	205	95.6%		12.7%	
Submucosa	406	90.4%		16.5%	
Differentiation type			0.020		0.192
Well differentiated	327	94.5%		13.5%	
Undifferentiated	284	89.4%		17.3%	
Lesion location			0.368		0.448
Lower cancer	284	93.0%		14.4%	
Middle cancer	241	92.5%		14.5%	
Upper cancer	86	88.4%		19.8%	
Tumor diameter			0.378		0.750
<1 cm	108	94.4%		13.9%	
1–3 cm	287	92.7%		14.6%	
>3 cm	216	90.3%		16.7%	
Macroscopic type			0.087		0.670
Elevated type	116	95.7%		13.8%	
Flat type	153	94.1%		13.7%	
Depressed type	342	90.1%		16.4%	
Morphological ulceration			0.031		0.371
No	269	94.8%		13.8%	
Yes	342	90.1%		16.4%	
LVI			0.046		0.354
No	566	92.8%		14.8%	
Yes	45	84.4%		20.0%	
Serum CEA			0.589		0.614
<5 ng/ml	560	92.3%		15.0%	
≥5 ng/ml	51	90.2%		17.6%	
LNM			0.001		0.001
No	489	94.9%		12.5%	
Yes	122	81.1%		26.2%	
Retrieved LN			0.031		0.027
<15	218	89.0%		20.2%	
≥15	393	93.9%		13.3%	

LVI, lymphovascular invasion; CEA, Carcinoembryonic antigen; LNM, lymph node metastasis; LN, lymph nodes.

There were 105 relapses (17.2%) during the follow-up, and the overall 7-year relapse rate was 15.2%. Univariate analysis revealed that age, sex, the depth of tumor invasion, differentiation type, tumor location, tumor diameter, macroscopic type, morphological ulceration, LVI, and serum CEA were not related to the 7-year relapse rate (*P* > 0.05, **Table 5**), while regional LNM and retrieval of at least 15 lymph nodes were related to the 7-year relapse rate (*P*< 0.05, **Table 5**). Therefore, the risk of a 7-year relapse rate was diminished for patients with no LNM or for those in whom at least 15 lymph nodes could be retrieved.

## Discussion

Gastric cancer is a disease of high heterogeneity throughout the world. It has a poor prognosis of GC, as the 5-year survival rate is lower than 30% (2). One of the reasons for this poor prognosis is the advanced stage of the disease at the initial diagnosis (2). However, the prognosis of EGC is satisfactory, with a 5-year survival rate tending to be >90% (5). Recently, the resection range of EGC has been minimized, while cancer recurrence and overall survival in some patients should be given high status. The patients with LNM had a relatively higher recurrence rate and poorer survival rate than those without LNM (11). To improve the survival rate of patients with EGC, it is important to study the rule of LNM and use this rule to select appropriate surgical methods.

### LNM in EGC Patients

Lymph node metastasis is closely related to the prognosis of patients with EGC. For patients with LNM, radical gastrectomy is still the most effective treatment (15). Radical resection and standardized lymphadenectomy can be used for local radical resection and accurate pathological staging. Because of the national early cancer screening policy of digestive tract malignancy, improved health awareness of urban and rural residents, and improved gastroscopy techniques, an increasing number of EGC cases have been detected and diagnosed in a timely manner. The presence of LNM in EGC directly affects patients’ prognosis and is a key factor that influences the choice of treatment and prognosis. Pereira et al. (16) reported that the rate of LNM in EGC is 5.7–19.1%. The rate of LNM in this study was 20.0%, which was higher than that reported in the literature. A possible reason was that this group of patients generally underwent a large range of lymph node dissection.

Previous studies have reported that the rate of LNM in EGC is closely related to the depth of tumor invasion, which is 0–7% for intramucosal cancers and 10%–25% for submucosal cancers (17, 18). In this study, the rate of LNM was 14.1% (29/205) for mucosal cancer and 22.9% (93/406) for submucosal cancer (*P* < 0.05). To a certain extent, the depth of invasion and tumor diameter reflects the length of time from the initiation of cancer to the diagnosis, that is, the larger the diameter and the deeper the invasion of the tumor, the greater the chance of LNM. Our retrospective analysis showed that the rate of LNM was 14.8% (16/108) for tumor diameter <1 cm, 17.8% (51/287) for tumor diameter 1–3 cm and 25.5% (55/216) for tumor diameter >3 cm (*P*< 0.05). Jeon et al. (19) found that tumor macroscopic type and differentiation type were independent risk factors affecting LNM in EGC, and our study reached the same conclusion. It is worth noting that the 46.5% (284/611) of undifferentiated adenocarcinoma (poorly differentiated adenocarcinoma, undifferentiated adenocarcinoma, mucinous adenocarcinoma, and signet ring cell carcinoma) in this study was much higher than the 10–30% reported in previous studies (19), but it still suggested that undifferentiated GC may be one of the characteristics of EGC in China. In this study, there were 116 cases (19.0%) of elevated type, 153 cases (25.0%) of superficial type, and 342 cases (56.0%) of depressed type, and the rate of LNM in these cancer types was 14.7% (17/116), 15.7% (24/153) and 23.7%(81/342) respectively (*P*< 0.05). The rate of LNM depressed type was higher than that of the elevated and superficial types; most of the depressed type cases with LNM showed invasion of the muscular mucosa, which not only brought the cancer cells closer to the submucosa but also made it possible for the cells to metastasize through the capillaries and lymphatics in the muscular mucosa.

In a study including 506 Japanese EGC patients who did not meet the criteria for endoscopic mucosal ablation, Kawata et al. (20) concluded that LVI was the only independent risk factor for LNM. However, many factors affected the lymph node metastasis of EGC. In our research, the depth of tumor invasion, degree of tumor differentiation, tumor diameter, macroscopic type, morphological ulceration, and LVI were related to LNM in the univariate analysis (*P*< 0.050). The results of multivariate analysis showed that the depth of tumor invasion, the degree of tumor differentiation, morphological ulceration, and LVI were the independent risk factors for LNM in EGC (*P*< 0.050).

### Choice of EGC Treatment

The treatment methods of EGC include traditional open radical gastrectomy, laparoscopic-assisted radical gastrectomy, endoscopic mucosal resection (EMR), or endoscopic submucosal dissection (ESD). Standard radical mastectomy is still the optimum treatment choice. The rational choice of EGC treatment is mainly based on the accurate assessment of tumor growth, invasion range, differentiation type, macroscopic type, and LNM status before and during surgery. However, LNM status remains the key factor for surgical scheme. Overall survival was explicitly worse in patients with LNM than those without LNM, with a 5-year survival rate of 81.1% and 94.9%, respectively. Furthermore, the 5-year year survival rate was 88.5% for tumors with 1–2 positive nodes, 64.3% for tumors with 3–5 positive nodes, and 41.8% for tumors with >6 metastatic nodes. Furthermore, considering cancer relapse, the overall 7-year relapse rate was 15.2%, which was higher than that reported in previous studies (12). The high incidence rate of undifferentiated adenocarcinoma [46.5% in this study *versus* 10–30% in previous studies (19)] may be a contributing factor. Interestingly, the 7-year risk of relapse diminished for patients with no LNM or for those in whom at least 15 lymph nosed were retrieved, which was similar to a previous study (12). Therefore, the status of lymph nodes is crucial to the relapse or mortality risk of EGC patients. In this study, the independent risk factors for LNM were depth of invasion, morphological ulceration, LVI, and differentiation type. Hence, for patients with high risk factors of LNM, dissection of at least 15 lymph nodes and D2 radical operation were recommended in this study. However, for patients with low or even no risk of LNM, conventional surgical treatment is appropriate, even endoscopic resection, because the postoperative survival rate of endoscopic resection was not significantly different from that of patients undergoing open radical gastrectomy, which improved the postoperative quality of life (21). In the 2014 Japanese gastric cancer treatment guidelines, the Japanese Gastric Cancer Society defined intramucosal carcinoma, tumor diameter <2 cm, medium-to-high differentiation, and no morphological ulceration as the absolute indications of endoscopic mucosal dissection, believing that LNM is rare in such patients (22).

Furthermore, OS was definitively worst in patients with group 2 LNM. This research suggested that extended lymph node dissection is recommended (D2+) in EGC with morphological ulceration or disease located in the proximal third of the stomach because of its high rate of group 2 metastasis, which was similar to previous research (5). The lymphatic drainage of the stomach is parallel to the vascular system, and the lymphatic flow in the upper-third region usually accompanies the left stomach and splenic blood vessels. Lymph nodes surrounding these blood vessels are included in the second or more categories. This may explain why the risk of metastasis to group 2 lymph nodes is also significantly different owing to the longitudinal position of the tumor.

### Analysis of EGC Lymph Nodes and Prognosis

The results of univariate analysis of this research showed that the depth of tumor invasion, differentiation type, morphological ulceration, LVI, and regional LNM were related to postoperative survival; multivariate analysis showed that regional LNM is an independent risk factor affecting the prognosis of EGC patients. The 5-year survival rate of patients with EGC was 94.9% in the no LNM group and 81.1% in the LNM group. The prognosis of patients without regional LNM was far better than those with LNM in EGC. Obviously, regional LNM was the main prognostic factor for patients with EGC. Kunisaki et al. (6) analyzed 1,169 patients with EGC who underwent surgery: 1,052 patients without LNM had a 5-year survival rate of 99.1%, and 117 patients with LNM had a 5-year survival rate of 90.8%. Suzuki et al. (23) reported that the 5-year survival rate of lymph nodes with and without metastasis for EGC endoscopic submucosal dissection was 92.6% and 99.9%, respectively. The 5-year survival rate was 85.4% for patients with 1–2 positive nodes after EGC, and 62.3% for patients with >3 metastatic nodes. Given the poor prognosis of EGC patients with LNM, comprehensive treatment and rigorous follow-up should be conducted for these patients after surgery.

In summary, this study suggested that tumor invasion depth, differentiation type, morphological ulceration, and LVI were independent risk factors for EGC LNM. Clinicians should conduct a comprehensive analysis based on the above characteristics and choose a reasonable treatment method. Minimally invasive technology can be used in patients with EGC after reasonable selection, but to ensure relapse prevention and extend survival, patients with EGC with high risk factors for LNM should also undergo radical lymphadenectomy. The depth of tumor invasion, differentiation type, tumor type, LVI, and regional LNM are related to postoperative survival. Multivariate analysis showed that regional LNM was an independent risk factor that affects the prognosis of patients with EGC. The EGC patients with LNM were treated with comprehensive treatment and followed up closely. Moreover, for non-surgical patients with EGC, the significance of lymph nodes can also play an important role in chemotherapy and radiotherapy.

## Data Availability Statement

The original contributions presented in the study are included in the article/**Supplementary Material**. Further inquiries can be directed to the corresponding author.

## Ethics Statement

The studies involving human participants were reviewed and approved by Ethics Committee of Hunan Cancer Hospital and other five hospitals. Written informed consent for participation was not required for this study in accordance with the national legislation and the institutional requirements.

## Author Contributions

CZ and SLei conceived and designed the experiments. JW, LW, SLi, and FB carried out the main experiments and analyzed the data. HX, HS, ZL, and XT collected data. XT and HT helped design the experiments. CZ wrote the paper. SLei performed language correction. All authors contributed to the article and approved the submitted version.

## Funding

The study was supported by the Department of Finance of Hunan Province Technology Project (2050205, to CZ).

## Conflict of Interest

The authors declare that the research was conducted in the absence of any commercial or financial relationships that could be construed as a potential conflict of interest.

## Publisher’s Note

All claims expressed in this article are solely those of the authors and do not necessarily represent those of their affiliated organizations, or those of the publisher, the editors and the reviewers. Any product that may be evaluated in this article, or claim that may be made by its manufacturer, is not guaranteed or endorsed by the publisher.
