# Supplementary material for: Risk Factors of Lymph Node Metastasis and Its Prognostic Significance in Early Gastric Cancer: A Multicenter Study
Source: Front Oncol. 2021 Oct 13;11:649035. doi: 10.3389/fonc.2021.649035 (PMC8548692; doi:10.3389/fonc.2021.649035)
Supplement: Supplementary file 1 [file Table_1.docx]

**Supplementary table 1. The relationship between TNM stages and** **group 1&2 lymph node metastasis in EGC.**

| **Clusters** | Group 1 LNM (%) | Group 2 LNM (%) | *P* |
| --- | --- | --- | --- |
| Tumor diameter <1 cm |  |  | 0.541 |
| Stage Ⅰ | 10(90.9%) | 1(9.1%) |  |
| Stage Ⅱ~Ⅲ | 4(80.0%) | 1(20.0%) |  |
| Tumor diameter 1–3 cm |  |  | 0.287 |
| Stage Ⅰ | 29(80.6%) | 7(19.4%) |  |
| Stage Ⅱ~Ⅲ | 10(66.7%) | 5(33.3%) |  |
| Tumor diameter >3 cm |  |  | 0.080 |
| Stage Ⅰ | 29(74.4%) | 10(25.6%) |  |
| Stage Ⅱ~Ⅲ | 8(50.0%) | 8(50.0%) |  |

Abbreviations: LNM, lymph node metastasis.
